# A qualitative exploration of tuberculosis patients who were lost to follow-up in Malaysia

**DOI:** 10.1371/journal.pone.0289222

**Published:** 2023-09-07

**Authors:** Peter Seah Keng Tok, Li Ping Wong, Su May Liew, Asmah Razali, Mohd Ihsani Mahmood, Thilaka Chinnayah, Lisa Kawatsu, Haidar Rizal Toha, Khalijah Mohd Yusof, Rozanah Abd Rahman, Shaharom Nor Azian Che Mat Din, Tharani Loganathan

**Affiliations:** 1 Institute for Clinical Research, National Institutes of Health (NIH), Ministry of Health Malaysia, Shah Alam, Selangor, Malaysia; 2 Centre for Epidemiology and Evidence-Based Practice, Department of Social and Preventive Medicine, Faculty of Medicine, University of Malaya, Kuala Lumpur, Malaysia; 3 Department of Primary Care Medicine, Faculty of Medicine, University of Malaya, Kuala Lumpur, Malaysia; 4 Sector of TB/Leprosy, Disease Control Division, Ministry of Health Malaysia, Putrajaya, Malaysia; 5 Department of Epidemiology and Clinical Research, the Research Institute of Tuberculosis, Japan Anti-tuberculosis Association (RIT/JATA), Tokyo, Japan; 6 Johor State Health Department, Ministry of Health Malaysia, Johor Bahru, Johor, Malaysia; 7 Respiratory Medicine Department, Hospital Sultanah Aminah, Ministry of Health Malaysia, Johor Bahru, Johor, Malaysia; New Delhi Tuberculosis Centre, INDIA

## Abstract

**Background:**

Loss to follow-up (LTFU) is an unsuccessful treatment outcome for tuberculosis (TB) patients. In Malaysia, LTFU affects around 1 in 20 TB patients. Integration of qualitative research methods and evidence will provide a better understanding of LTFU and its underlying issues. In this study, we qualitatively explored TB patients’ experiences in receiving treatment and their reasons for leaving TB care.

**Method:**

In-depth interviews of 15 patients with a history of LTFU were conducted from January to September 2020. Interview guides were developed to explore TB patients’ experiences while receiving treatment, including challenges faced and reasons for treatment interruption. Data were thematically analysed using the framework method.

**Results:**

We identified 11 emerging themes that occurred at four levels of interaction with TB patients. First, at the patient personal level, TB beliefs referring to patients’ perception of illness and wellness, patients’ perceived role of traditional and complementary medicine, and substance abuse were important. Second, the healthcare system and treatment factors that were highlighted included the organisation of care and treatment, interaction with healthcare professionals, particularly in communication and counselling, and TB medications’ side effects. Third, structural factors including financial burden, logistical and transportation issues and work-related factors were identified to be barriers to treatment continuation. Fourth, the interpersonal level interaction of patients should not be neglected; this includes family relationships and support as well as peer influence.

**Conclusion:**

Study findings put forth issues and challenges faced by TB patients while receiving treatment and underscore areas where actions can be taken. This will contribute to informing the development and implementation of future TB control strategies that are responsive to TB patients’ needs and concerns, to effectively address LTFU and ensure better treatment completion rates among TB patients in Malaysia.

## Introduction

Tuberculosis (TB) remains a major public health problem globally, affecting 10 million people and contributing to 1.5 million deaths in 2018 [1]. The End TB strategy led by the World Health Organization (WHO) proposes to “end the global TB epidemic” by 2035 [2]. One of the key indicators to monitor the implementation of this strategy at a country level is the TB treatment success rate. The recommended target level for TB treatment success rate is ≥90% by 2025 at the latest [1].

Malaysia has an intermediate TB burden, with an estimated incidence rate of 92 (range: 79–106) per 100,000 population in 2018 [1]. The TB control program in Malaysia has expanded over the years, with a focus on early detection of TB cases, ensuring quality laboratory services, development and conduct of training modules and guidelines, usage of a standardized TB recording and reporting system, and strengthening collaboration with other agencies [3]. Efforts to standardise the management of TB cases at all levels of patient care in Malaysia with the aim of improving patient care were emphasized by the development of an evidence-based clinical practice guideline for the management of TB, which provides recommendations for investigations and treatment of TB (in adults, children, during pregnancy, TB-HIV co-infection), including the follow-up and management of adverse TB drug events [4]. A patient-centred case management approach, including the practice of directly observed therapy (DOT), is strongly recommended when managing persons with TB, with data showing DOT being practised for almost 90% of all TB cases in 2015 in Malaysia [3].

Although significant progress has been made over the years toward improving treatment outcomes of TB cases in Malaysia, the TB treatment success rates remained below the recommended target of ≥90%. In the recent cohort analysis using national TB registry data in Malaysia involving TB patients from 2014 until 2017, the overall TB treatment success rate was observed to be 80.7% among drug-susceptible TB patients (TB cases with drug resistance were excluded) [5]. Among the 19.3% who had unsuccessful outcomes, 10.2% succumbed due to TB, 5.3% were lost to follow-up, 3.6% had outcomes not evaluated, and the remaining (<1%) failed treatment.

Loss to follow-up (LTFU) refers to TB patients who did not start TB treatment or whose TB treatment was interrupted, for a duration of two consecutive months or more [6]. Worldwide, existing literature on TB has described over 200 variables associated with LTFU [7]. These include a spectrum of patients’ sociodemographic characteristics, medical co-morbidities, risk behaviours such as alcohol consumption and illicit drug abuse, as well as their TB disease clinical variables [8–16]. However, the association between these quantitative variables were not consistent across studies, suggesting that the underlying factors contributing to LTFU are likely to be context-specific. In addition, most of the characteristics investigated often relate poorly to TB patients’ intention and motivation to comply with or complete their treatment regimen [7]. There is therefore a need to integrate qualitative evidence to provide a better understanding of LTFU and its underlying issues. WHO also reiterated that research into human behaviour should encompass a wide range of enquiry approaches, including qualitative and quantitative methods [7]. In the context of TB, this could be because treatment adherence behaviour is complex and cannot be fully explained by the routinely captured demographic, socio-economic or clinical factors in surveillance databases [17–19].

In Malaysia, there is a paucity of evidence investigating determinants of LTFU among TB patients, particularly at the national level. To the best of our knowledge, there has not been any prior qualitative evidence exploring LTFU and its underlying reasons in Malaysia. To address this gap, we conducted a qualitative exploration into TB patients’ experiences in receiving TB treatment, focusing on the issues and challenges faced, and the reasons leading to treatment interruption, through in-depth interviews with TB patients in Malaysia who had a history of LTFU.

## Materials and methods

Qualitative methods were used in this study. This study is part of a national-level project to evaluate determinants of TB treatment outcomes in Malaysia. The investigation for determinants of unsuccessful treatment outcomes in general and all-cause mortality has been published previously [5]. This paper is the first investigation into the LTFU outcome, using a qualitative approach.

### Definition of terms

The WHO’s definitions and reporting framework for TB standardise the definitions and reporting structures for TB cases, including TB treatment outcomes [6]. The list of TB treatment outcomes includes (1) cured, (2) treatment completed, (3) treatment failed, (4) died, (5) LTFU and (6) not evaluated (including transferred out). This list of treatment outcomes is assigned to all bacteriologically confirmed and clinically diagnosed TB cases who were treated for drug-susceptible TB (patients treated for drug-resistant TB using second-line treatment follow different definitions and are excluded from the scope of this study). LTFU refers to “a TB patient who did not start TB treatment or whose TB treatment was interrupted, for a duration of two consecutive months or more”.

### Study population

Study participants were recruited from healthcare facilities in the state of Johor, Malaysia. Johor is a state in the southern part of Peninsular Malaysia. According to the national census conducted in 2010, it is the second-most populous state in Malaysia, with a population of 3.35 million people (11.8% of 28.3 million people total in Malaysia) [20]. Findings from the census indicated good representativeness of the Johor state population with the national average in Malaysia with regards to the sex ratio, age groups, ethnic composition, and urbanisation level. In 2015, Johor state contributed 9.9% (2,409 cases out of a total of 24,220 cases) of total TB cases in Malaysia, with a TB notification rate of 67.8 per 100,000 population (national average: 79.4 per 100,000) [3]. During the same year, there were 135 reported TB deaths in Johor state or a TB mortality rate of 3.8 per 100,000 population (national average: 5.5 per 100,000 population) [3].

### Sampling and recruitment

Participants were TB patients aged 18 years and above who had fulfilled the criteria of LTFU (refer to ‘Definition of terms’ above) on at least one prior occasion throughout their TB treatment regimen(s). We purposively recruited eligible TB patients in Johor by identifying eligible patients from the national electronic TB surveillance database. TB is a notifiable disease in Malaysia, and the capture of routine surveillance data for TB patients had been previously described [5]. In this study, TB patients who were treated for drug-resistant TB were excluded as they follow different TB treatment regimens and follow-up periods (including longer duration of TB treatment) [4, 6].

From the list of eligible TB patients, a maximum variation sampling strategy was adopted to include participants that cut across various socio-demographic and TB disease characteristics. The study sample size was not pre-determined; study participants were consecutively recruited until thematic saturation was reached, and investigators agreed that further interviews would not yield additional information.

### Data collection

Data collection was conducted from January to September 2020. We conducted 15 in-depth interviews with 15 TB patients (one interview per patient). All interviews were conducted in person in Johor on an individual basis. Potential study participants were invited to participate by telephone calls, and interviews were scheduled and conducted at either a nearby healthcare facility or at TB patients’ places of residence, according to the patient’s preference. All potential participants approached consented to participate and completed the interviews. Participants’ characteristics are shown in Table 1.

**Table 1:** Characteristics of the study participants (n = 15).

Characteristics	*n* (%)
Mean age, years (SD)	37.6 (13.86)
Age range, years	21–67
Gender	
Female	3 (20.0)
Male	12 (80.0)
Nationality	
Malaysian	15 (100.0)
Non-Malaysian	0 (0.0)
Location of residence	
Urban	11 (73.3)
Rural	4 (26.7)
Education level (highest)	
Tertiary	1 (6.7)
Secondary	11 (73.3)
Primary	2 (20.0)
Diabetes mellitus	
Yes	2 (13.3)
No	13 (86.7)
Smoking	
Yes	8 (53.3)
No	7 (46.7)
TB site (of infection)	
Pulmonary TB	12 (80.0)
Extrapulmonary TB	3 (20.0)
Sputum smear	
Positive	12 (80.0)
Negative	3 (20.0)
HIV infection	
Positive	1 (6.7)
Negative	14 (93.3)

HIV, human immunodeficiency virus; SD, standard deviation; TB, tuberculosis.

Semi-structured interview guides were developed to explore TB patients’ experiences throughout receiving their TB treatment, including challenges faced, and the underlying reasons for treatment interruption. The interview guides were constructed based on themes identified *a priori* from a review of existing literature and input from experts and stakeholders in TB control and prevention in Malaysia. This included experts in qualitative research methodology, technical staff in the field involved in clinical TB case management, and healthcare personnel involved in providing care and follow-up for TB patients. The study team then discussed and refined the interview guides. See S1 File for interview guides.

Interviews averaged from 30 minutes to 1 hour in length and were conducted in either English or *Bahasa Malaysia* (Malay language), according to the patient’s preference. All interviews were audio-recorded and transcribed verbatim for analysis. Data collection and analysis were conducted continuously in an iterative manner. This concurrent analysis also informed, when necessary, further refinement of the interview guide throughout the data collection period.

### Data analysis and reporting

Data analysis was performed in an exploratory and inductive manner, with regular discussion amongst the investigators. Data analysis was performed after each interview, and findings were compared and consolidated with those of previous interviews. Interview data were analysed using thematic analysis, specifically the framework method [21, 22]. In this method, the investigators first carefully read the transcripts for familiarisation before initiating the coding process. Next, codes were assigned to parts or excerpts of the transcripts interpreted as important. The codes were then compared, consolidated, organised, and grouped into categories (or themes), forming a working analytical framework. Further transcripts were then coded and reviewed, and additional codes or categories relevant to the study objective were added to enrich the framework matrix. Finally, the framework matrix was populated with summarised data containing all the important codes and themes.

Interview transcripts were converted into rich text format and imported into Nvivo 12 Pro software (QSR International Pty Ltd, Doncaster, Victoria, Australia) for data analysis. For interviews conducted in *Bahasa Malaysia*, the transcripts were analysed in the same language, while extracted interview excerpts were translated into English for the reporting of the study findings. This study is reported following the Standards for Reporting Qualitative Research (SRQR) guideline [23] (S2 File).

### Reflexivity

The lead investigator (PSKT) who conducted all the interviews in this study was a medical doctor with a background in clinical research and public health and had extensively reviewed the existing literature on LTFU among TB patients. To reduce potential bias in the data collection and analysis, interview guides were prepared and used during the interviews. These guides considered and gathered input from existing literature and relevant experts (see S1 File for more details). For data analysis, other investigators were involved to ensure dependability. Any disagreements with the analysis were regularly discussed, and a consensus was reached at the investigators’ meeting. The lead investigator was not involved in the clinical management of TB patients and was not affiliated with the respective healthcare facilities where the TB patients were receiving care from.

### Ethics consideration

Participant information sheets containing detailed study procedures including the conduct of interviews and audio-recording of interviews, participants’ rights and responsibilities, potential risks and benefits, and patients’ confidentiality and usage of study data, were distributed and explained to every potential study participant in either English or *Bahasa Malaysia*. Written voluntary informed consent was obtained from all participants before the conduct of any data collection for this study. All participants were informed and aware that study participation was entirely voluntary, and that they may withdraw their participation at any time. They were also free to refuse to answer any questions during the interviews and may opt to terminate the interviews at any point. All participants agreed to be audio recorded and quoted anonymously in publications. No personal information or identifiers were collected, maintained, or analysed throughout the study, and participants were quoted anonymously in interview transcripts and publications. All study participants were compensated for their time and travel expenses (when applicable), with the honorarium amount reviewed and approved by the ethics committee.

This study was registered in the National Medical Research Register (NMRR-18-3399-45073). The study protocol was reviewed and approved by the Medical Research and Ethics Committee (MREC), Ministry of Health, Malaysia.

## Results

There were altogether 11 themes that emerged from the in-depth interviews conducted in this study. The themes were grouped into four main groups according to the level they occur or interact with TB patients: patient-level, structural, healthcare system and TB treatment, and interpersonal level interaction. Table 2 summarises the main groups and themes emerging from this study.

**Table 2:** Main findings of the study.

Groups	Themes
**Patient-level factors**	TB beliefs: perception of illness and wellness
	Role of traditional and complementary medicine
	Substance abuse
**Healthcare system and TB treatment factors**	Healthcare system: organisation of care and treatment
	Healthcare professionals: communication and counselling
	Medication side effects
**Structural factors**	Financial burden
	Logistic/transportation issues
	Work-related factors
**Interpersonal level interaction**	Family relationships and support
	Peers’ influence

TB, tuberculosis.

### Patient-level factors

#### TB beliefs: Perception of illness and wellness

Patients’ perception of TB disease was explored in the interviews conducted, with most of the TB patients able to correctly describe TB as an infectious illness with potentially fatal complications. There were some patients, however, who acknowledged that they do not fully understand TB, apart from it being infectious and associated with respiratory symptoms. This patient, who had extrapulmonary TB, initially did not believe her diagnosis as she was not aware that TB can present with extrapulmonary involvement.

"*Honestly*, *I do not even know about this disease*. *I know during schooling time*, *we learned (when you have) TB… cough… like that only*. *After finish schooling*… *the doctor confirmed that I had TB*. *But I do not believe*… *because*, *I know that TB is cough*. *But for me*, *the problem is near my stomach [points toward abdominal cavity]*… *like that*.*" (24 years old*, *woman)*

Although the patients interviewed had a varying understanding of TB, all of them accepted the diagnosis and started taking anti-TB medications. However, almost half of them divulged that they gradually started to skip their follow-up visits after some time, followed by stopping the medications altogether when they feel better, or when their symptoms resolved. This is because they felt that they were already cured upon symptom resolution and thus no longer needed the medications.

"*I feel*, *like*, *no need (the medications) … because*, *I am back*, *I feel like I am back to normal*.*" (29 years old*, *man)*

#### Role of traditional and complementary medicine (TCM)

Among the patients interviewed, some mentioned that they also took TCM and expressed that it helped to provide symptomatic relief and was complementary to the anti-TB medications. TCM modalities mentioned included traditional herbs and therapeutic massages.

"*When eating village medications*, *like… raw plants*, *leaves*, *vegetables*, *fruits*, *oranges*, *okay*. *Body all back to normal*. *I still have a cough*, *but no phlegm*. *Clear*.*" (27 years old*, *man)*

"*Like… just… we go for a massage*. *Massage for body*, *for chest*, *what not… we check*. *That is… treatment*, *traditional treatment*, *with hospital treatment*, *balanced*.*" (27 years old*, *man)*

However, the role of TCM took a different course for some TB patients. Some regarded TCM as supplementary, but some patients preferred TCM and regarded it as an equivalent, eventually replacing the anti-TB medications. This patient added that TCM should be offered as an option for all TB patients.

"*For us*, *we do it our way*. *Our own way of treatment*. *At the same time*, *we let you know*. *Inform the hospital side*. *That is according to me*. *Just… okay*, *we give how much time… for example*, *two months*, *or three months for healing*, *for our own way*. *Do it (our own way)*.*" (34 years old*, *woman)*

#### Substance abuse

There were several patients interviewed who had experience with substance abuse. Two patients interviewed noted that their dependency on illicit substances during their TB treatment regime contributed to treatment interruption and eventually to their discontinuation of the anti-TB medications. This patient explained that when he was dependent on the illicit drugs he was taking, he could not commit to taking anti-TB medications regularly.

"*First*, *I give up taking medications (referring to anti-TB medications) every day*, *every day I take (illicit) drugs (instead)*. *Because when we depend on these drugs*, *then we take medications*, *and we smoke back the drugs… already*, *no feel*, *uh… feel towards the drug" (32 years old*, *man)*

### Healthcare system and TB treatment factors

#### Healthcare system–organisation of care and treatment

TB patients interviewed voiced their grievances about the organisation of TB care treatment which made it difficult for them to adhere to their TB treatment regimens. In particular, the rigid implementation of the directly observed therapy (DOT), which is used in TB clinics in government healthcare facilities in Malaysia to administer TB medications to TB patients. Most of the patients expressed that it was challenging to meet the DOT requirements, especially the need to be physically present (often daily) at the TB clinics to receive medications. Additionally, the limited working hours of the TB clinics administering DOT and the inflexibility to allow temporary provisions (for example, caretakers to collect medications on their behalf, transfer temporarily to another clinic for convenience, or allow an advance dispensation of a week or two’s medications during emergencies were also mentioned as barriers towards maintaining the continuity of TB treatment.

“*After that*, *the final month of treatment was near Chinese (Lunar) New Year*. *I did come here for a check-up with the doctor*. *I have asked the doctor*, *‘… this month*, *I am a little busy*, *because there are some problems at my work*.*’ So*, *I asked*: *‘Can I transfer the place… the clinic to take medications*? *Because I know my own time*, *I want to recover*, *I do not want to skip (medication) right*?*’ So*, *I have asked the doctor*, *(but) the doctor said cannot… because if everyone asked like this*, *they cannot accommodate (the request) … very busy*.*" (47 years old*, *man)*

"*Okay*, *every day actually*, *I did trouble my husband as well*. *(I) have to fast during the fasting month*. *When he (the patient’s husband) came back from work*, *I asked him to take(collect) the medications*. *At that time*, *I already stopped working*. *I asked him to take the medications (on my behalf)*. *But the nurse scolded (him) at the clinic*. *I do not know*, *the nurse is always like that… sometimes if I asked my husband to take it*, *she will scold (him)*. *But here (patient changed DOT location after restarting treatment)*, *the nurse is okay*.*" (34 years old*, *woman)*

Other issues mentioned regarding the organisation of TB care and treatment included long waiting times–this included waiting times for an initial appointment for a consultation, during subsequent clinic visits, and even during the daily DOT sessions. One patient mentioned that the doctors attending to him during follow-up consultation sessions were always different, leading to concerns about the quality and consistency of the TB treatment given.

"*Different doctor… the specialist here always changes*, *so in my heart*, *I was asking*, *how do you (as a doctor) treat if the patients always change*, *rotated around… right*? *(57 years old*, *man)*

#### Healthcare professionals: Communication and counselling

The interaction between TB patients and healthcare professionals was frequently mentioned during interviews. Several TB patients interviewed recalled unpleasant encounters with healthcare professionals, which subsequently demotivated them from continuing the TB treatment. Some of them expressed regret that their complaints were often not taken seriously and wished that the attending healthcare professionals could put in more effort and attention to their concerns. For example, patients felt that the doctors were not empathetic enough when they complained about the side effects of TB medications. Patients also felt that doctors were unwilling to discuss alternative options for medications.

"*The only thing I am not satisfied (with) is that some doctors*, *sometimes… like when we are talking now*,*… it is like he hears*, *the thing goes in here and goes out there [the patient pointed his hand towards his left ear*, *followed by his right ear] … uh*, *like that*. *What we told is not very important*, *for like us uncles*, *usually*, *uncles who may not be educated*, *my view is more or less like that*. *Right*? *Maybe if there is someone with position or rank*, *maybe the doctor will be more serious*, *right*? *(57 years old*, *man)*

"*Just that*, *sometimes… we as patients*, *we also want our doctors to understand us*. *I mean… try*, *let us both find a way together*. *Medications*, *we discuss together*.*" (34 years old*, *woman)*

#### Medication side effects

Undesirable side effects of the TB medications and their impact on patients’ daily lives, including work, were remerged as a major contributory factor towards treatment disruption. At least half of the patients interviewed attributed medication side effects as a crucial factor in their decision to stop TB treatment. The common medication side effects mentioned include fatigue, feeling tired or weak, joint pains, dizziness, drowsiness, gastrointestinal symptoms (loss of appetite, nausea, vomiting), rashes and itchy skin.

"*The pain from medications starts from the head*. *The medications even (caused pain) until we are like… lost*, *lost our mind*. *Like you do not know what is happening around you*. *Just raise your hand*, *want to talk*, *but do not know what to say*. *That last one was scary*. *So*, *I avoided coming to see the doctor*. *When I take the medications*, *I will collapse for two or three days*.*" (57 years old*, *man)*

Several patients shared that they felt worse after they started consuming the TB medications. In other words, the medication’s side effects were at times more unbearable than the symptoms of the TB disease itself. One patient shared that the side effects from medications impacted his daily life and affected his ability to work.

"*To me*, *this TB disease… there is a cure to it*, *the medications*. *But the medications have an effect on our daily work*. *So*, *the TB medications will have an effect on (us)*. *We will become… ‘out’*. *Body becomes weak*, *to work*.*" (29 years old*, *man)*

### Structural factors

#### Financial burden

All the TB patients in this study were Malaysian citizens and were eligible to receive the anti-TB medications for free from government healthcare facilities. However, the financial burden was frequently mentioned as a major barrier to treatment continuation amongst those interviewed. The financial constraints were mainly due to the indirect costs involved, which included travel expenses to healthcare facilities and lost wages when the patients or their caretakers (family members) needed to take time off work to attend the clinic sessions.

"*For me to come here… there is no money for me to come here*, *to support me to come here*. *The heart (referring to the patient’s intention*, *figurative speech) wants to come for treatment*, *but no… (I) cannot afford it*.*" (48 years old*, *man)*

#### Logistic/Transportation issues

TB patients interviewed also lamented the unavailability of transportation means to travel to the nearest healthcare facilities to receive TB treatment. Some of the patients interviewed were dependent on family members or caretakers to bring them to the clinics, and others simply did not have any means of transportation to travel daily to healthcare facilities for the medications.

"*(I) live far away… so*, *as I said every day*, *I want to go in the morning…*, *(the) transport vehicle there is only one*. *My brother is also working*. *He does not have transport*. *So*, *sometimes he uses*, *sometimes I use*, *sometimes he uses*.*" (31 years old*, *man)*

While private taxis and e-hailing services are available, they are not sustainable over the long duration of TB treatment as the out-of-pocket expenses for travel soon became a financial constraint to patients.

"*When I call Grab (e-hailing service) … from my house to here (referring to the healthcare facility where the interview was conducted) right*, *five ringgits (Ringgit Malaysia*, *Malaysia’s currency) right*? *Five ringgits*. *If I go and come back (return trip)*, *it is already ten ringgits*. *Yes*, *not enough*, *right*? *Later*, *I want to eat at the workplace… not enough (money)*.*" (21 years old*, *man)*

#### Work-related factors

The majority of the TB patients interviewed expressed that their work was affected by the TB disease and the requirements for its treatment (at least six months duration). This included difficulties to accommodate travelling time to the healthcare facilities to receive TB medications. Often, this led to lost wages when patients needed to take time off work. This patient indicated that while he wanted to continue treatment, his work did not allow him to attend the daily sessions for DOT administration in TB clinics.

"*I skipped (treatment) last time because of my time and the clinic time… not merge*. *Because my working time… when I go back (from work)*, *the clinic is already closed*.*" (47 years old*, *man)*

Some TB patients explained that they lost their jobs due to this disease as they were physically unfit to work. One of the TB patients alluded to being discriminated against at her workplace, as she was asked to resign upon her TB diagnosis due to fear of possible transmission to other colleagues.

"*After that… assistant manager came*, *she talked to me*, *she said*, *‘… you cannot come today you know*, *later infect other people*.*’ What do you feel when you hear people say that*? *After that*, *I kind of felt down*. *After that*, *I called HR (human resources personnel)*, *HR was not at that place*, *HR was at another place*. *I called HR*. *HR said… you need to write a letter… resign within twenty-four hours*. *HR asked me to do it*. *Actually*, *I do not know*, *at that time I was thinking like*, *‘… they do not want me anymore*, *right*?*’ So*, *I did it (resigned as told)*.*" (24 years old*, *woman)*

### Interpersonal level interaction

#### Family relationships and support

In the interviews conducted, TB patients’ interpersonal level of interaction was also discussed, particularly regarding its potential impact towards the patients’ adherence to TB treatment. Amongst all interactions with others, family members were the most commonly mentioned. The relationships with family members, who often acted as caretakers when patients were ill, and the support from these family members, were often quoted as instrumental towards ensuring TB treatment continuation. There was one patient who felt affected by the lack of support from her husband and in-laws, who were indifferent and offered no help when she was unwell.

"*During the time I was on medications*, *all my joints were in pain*. *It is hard even for me to get up*. *When I am already seated down*, *I want to get up… (it was) difficult*. *So*, *how am I going to do all the work (referring to housework)*? *There is a limit*, *you know*. *I cannot do all the work; I need his help (husband)*. *But he is not the type that was accustomed to help with housework*.*" (24 years old*, *woman)*

Another TB patient who was bed-bound due to multiple co-morbidities was dependent on his mother to keep track of his clinic appointments, as he was not fit to do so. During the interview, he explained that considering his condition and dependency on others (especially his mother), it was challenging to keep up with follow-up visits.

"*Sometimes that date*, *only my mother knows*. *She also said*, *already forget (his mother forgot the appointment date)*. *Then for me*, *all the dates I forgot… my mother was late in reminding me*.*" (29 years old*, *man)*

#### Peers’ influence

Most TB patients interviewed did not attribute the significance of influence or interactions from the surrounding community to be impactful towards their TB treatment. However, several patients mentioned that their relationships with their peers were affected as a result of the TB disease. Most of the patients chose not to disclose their TB diagnosis to their peers or community openly, while some only divulged to persons close to them or whom they trusted.

"*Before I got TB*, *there was no problem*. *When I got this TB*, *… there is some problem with the interaction (with friends)*. *My friends also know about it*. *The relationship (amongst friends) is also out (over)*. *Until now"*. *(29 years old*, *man)*

There was one patient who mentioned that the remarks from his friends somewhat influenced his decision to stop TB treatment. At that time, the patient and his friends all were involved in substance abuse.

"*Drugs were more important than medications*. *If during that time… (I was) taking drugs (illicit substances)*, *I also take (anti-TB) medications*. *My friends also a lot of them said*, *you take TB medications (and) you also take drugs (illicit substances)*, *there is no use*. *Right*? *No use*. *So*, *I took a short-sighted decision*, *I chose drugs*.*" (32 years old*, *man)*

### Suggestions for improvement

In the interviews conducted, opinions and suggestions from the TB patients on what can be improved to the current system of TB treatment administration and follow-up were also discussed. The majority of patients indicated that the current TB medications administration using DOT should allow for greater flexibility and provisions for TB patients who often cannot come daily to the TB clinics for the entire six months duration (or more). Some of the suggestions were to consider giving weekly doses of TB medications (instead of daily) and to set up healthcare centres that operate outside of the normal working hours to allow working patients to receive the medications after their work.

" *… like medications*, *once a week*, *okay*? *This week’s medications*, *you take*. *In a month*, *check-up*. *We will go for a check-up*, *and the doctor will know…*, *like if the infection will be better or if it increases*, *or decreases… he will know*, *right*? *If we do not go*, *do not take the medications… he will know"*. *(48 years old*, *man)*

Some of the patients interviewed, particularly those who faced financial constraints, suggested assistance be given to cover out-of-pocket expenses, particularly transportation costs, for patients to be able to attend the scheduled DOT sessions in TB clinics.

" *… give allowance for transport*,*… so that the patient does not stop coming*. *We… give him an allowance*, *ask him to come using a taxi or … the government can give him some help or something…" (31 years old*, *man)*

Other suggestions included remarks on how communication and engagement by healthcare professionals can be improved. In particular, patients expressed their wishes for their opinions and complaints to be better acknowledged and discussed by the attending healthcare professionals.

"*Uh… doctor needs to hear*… *Sometimes the doctors they all… I do not know*, *maybe tension (stressed) or maybe sometimes they also do not want to hear what we say…" (47 years old*, *man)*

## Discussion

**Fig 1:**
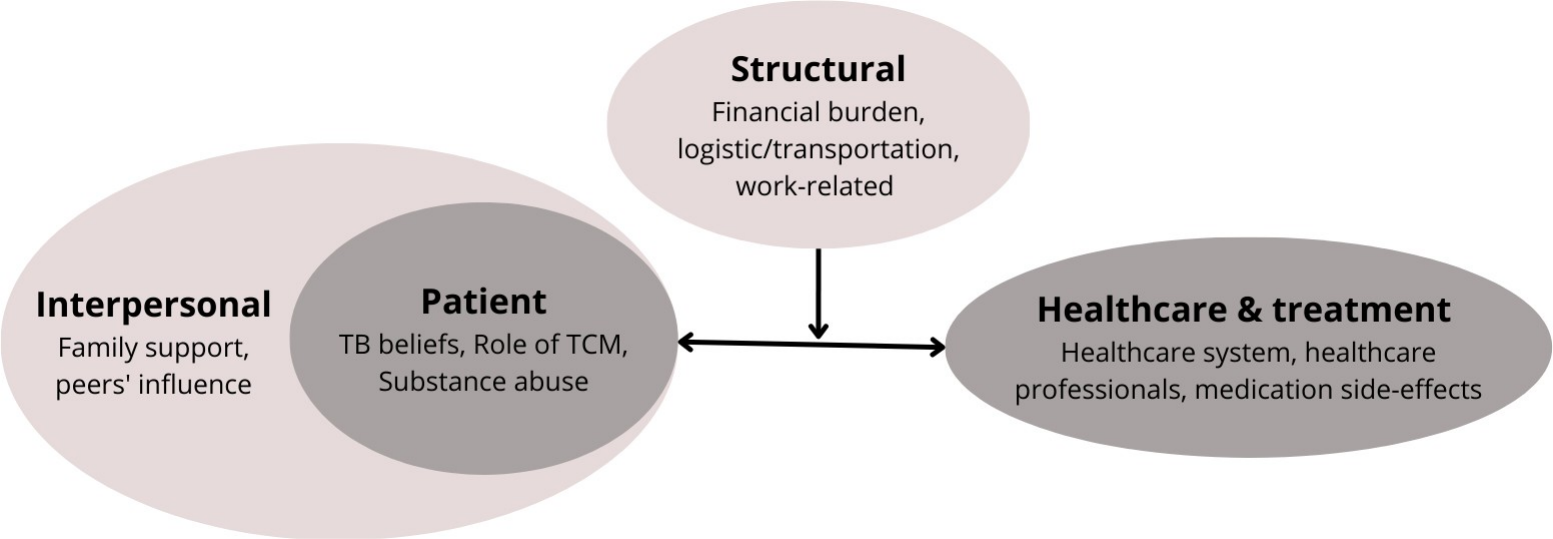
Summary and visualisation of study findings–four main groups of themes. TCM, traditional and complementary medicine; TB, tuberculosis.

Our study findings identified 11 themes underlying the issues and challenges faced by TB patients throughout their TB treatment regimens, and the factors leading to the patients interrupting or leaving TB care (thus fulfilling the LTFU treatment outcome definition). Most of the themes identified in this study were intricately related to one another and likely to contribute together or have collective effects in influencing TB patients’ treatment adherence and, subsequently, their decision on continuing or leaving TB care. The four main groups of themes, and how they relate to one another, are visualised in Fig 1 below.

The first level, intrinsically the patient’s personal level, demonstrated the importance of how TB patients view and regard their TB treatment–specifically, on whether they still regard the treatment as beneficial or the need to continue them throughout the whole duration as prescribed. We observed that when the patients felt better after a short period of receiving treatment (symptoms resolution), some of them interpreted this as being cured, and therefore do not appreciate the need to continue the TB medications any longer. Others may also substitute prescribed treatment with other forms of therapy, including traditional and complementary medicine modalities. Previous studies from other countries also attributed this perception of wellness or feeling better as one of the contributing reasons for LTFU among TB patients [24–26]. However, this is a worrying concern because poor adherence or incomplete TB treatment can contribute to drug resistance and prolonged infectiousness, during which they can still transmit the disease to others in their community [25, 27]. To address this, it would be prudent to look into the current TB medication counselling efforts, and where the appropriate emphasis should be made to educate on the importance of treatment completion despite symptom resolution, and the risks of incomplete treatment. Further, the delivery of such information or counselling should be consistent and reinforced throughout the entire duration of TB treatment.

The second level involved the other crucial component of TB treatment administration, being the healthcare system factors, including healthcare professionals and the TB treatment itself. In the organisation and delivery of TB care, the main discussion points were related to how patients had difficulties in meeting the rigid requirement of being physically present at TB clinics (often daily) to receive the TB medications, under the DOT approach. Work commitments, logistic and transportation issues as well as financial barriers to cover out-of-pocket travel expenses were cited as underlying challenges faced to meet the daily DOT requirements. To address this, it may be prudent to look into the current DOT practices for TB treatment administration and explore how they can be enhanced. A pertinent guiding principle, as mentioned in the clinical practice guidelines for the management of TB [4], is that DOT should be patient-centred, incorporating negotiations and taking into account patients’ characteristics and preferences.

Additionally, instances of unpleasant encounters with healthcare professionals including the occasional communication breakdown were also brought up and patients wished for better patient-provider rapport in the overall experience of receiving TB treatment. This is not entirely unexpected as in studies conducted elsewhere, the relationship between patients and treatment providers has also been observed to influence treatment adherence [25, 28–31]. Efforts to improve the patient-provider rapport and communication, therefore, remain crucial and should be consistently encouraged. Similarly, TB medication side effects as one of the contributory factors for stopping treatment in this study have also been widely accounted for in previous studies as one of the main reasons for LTFU among TB patients [17, 25, 26, 30, 32, 33]. The management and follow-up of adverse drug events for all TB patients in Malaysia, including the patients in this study, follow the recommendations outlined in the clinical practice guidelines for the management of TB [4]. The role of healthcare professionals is again important here, i.e., to recognize and acknowledge patients’ complaints on the medications’ side effects, and ensure responsive and appropriate actions are taken to address their discomfort, while at the same time, ensuring treatment compliance without compromising on the adequacy of the TB treatment given.

Thirdly, apart from patients’ personal factors and the healthcare system, we also identified certain structural factors that posed as potential barriers hindering TB patients from seeking and sustaining, the receipt of TB treatment over the entire duration prescribed. This included financial constraints, logistic and transportation issues, and work-related factors–all of which may relate closely with one another. Themes related to financial burden have also been reported in many studies worldwide [17, 25, 34, 35]. Efforts to address this should look into facilitating and considering the provision of assistance to patients who needed such help. This could be in the form of monetary incentives, which had been shown in several previous studies in different settings to be effective in improving treatment adherence and reducing LTFU rates [36–38]. Additionally, non-cash initiatives that could ease the financial burden of patients, including offering material incentives and enablers such as vouchers and transportation services to improve treatment adherence [39], may be explored for their feasibility and effectiveness in local settings.

Last but not least, as observed in our study findings, it is important to be cognizant that TB patients, like every other individual, live within a society and have interpersonal interactions with peers, family and the community around them. As such, their treatment adherence behaviour may be influenced by interpersonal relationships, as observed in our study findings. In particular, our study observed that good family relationships and strong support from family members, who were often the patients’ caretakers, were influential towards ensuring treatment continuation. Studies conducted elsewhere also identified family support as having a strong influence on patient adherence to treatment [17, 25, 40, 41]. The implication from this finding would be that in future, efforts on health promotion and education on TB treatment, or specific measures to promote adherence, should consider involving patients’ family members or caretakers.

The strength of our study is that it provides crucial input and evidence on reasons for LTFU among TB patients in Malaysia through patients’ own experiences. To the best of our knowledge, this is the first study that explored, in detail, the reasons for leaving TB care among TB patients in Malaysia, addressing the paucity of evidence in this area to inform and guide public health actions. Our study findings highlight the issues and challenges experienced by TB patients in Malaysia that should be given appropriate priority and attention. The important themes identified also represent leverage points where actions can be taken–for example, existing control efforts can be improved, or new policy decisions can be implemented.

Qualitative research is a powerful tool to provide profound insights into human behaviour and their experience of disease and treatment [42]. In the context of TB treatment, which is administered over a long duration, it is well recognised from existing literature that quantitative investigation of factors routinely captured in surveillance data is not sufficient to explain and understand treatment behaviour among patients [7, 17, 19, 25, 43]. The qualitative findings from this study, therefore, will be beneficial in addition and complementary to quantitative analysis of determinants for LTFU to provide a better understanding of LTFU among TB patients, as well as to guide the development and implementation of responsive and targeted strategies to address it.

Our study was not without its limitations. First, it was difficult to recruit study participants who cover all the variations of sociodemographic characteristics and clinical variables as the sampling pool of eligible patients was small and it was challenging to contact patients who were LTFU and had discontinued treatment. Of particular note, we did not manage to recruit patients who were non-Malaysians, and not many of them were females, had DM, or had TB/HIV co-infection (Table 1). Second, findings in this study did not differentiate between the timing of LTFU, or when it occurred, for patients interviewed. Third, the qualitative nature of the present study may not allow generalisation to TB patients in other contexts and settings. Finally, the scope of investigation in this study was limited to the TB patients’ point of view through their experiences in receiving TB treatment.

Moving forward, future research that investigates further into LTFU among TB patients should be encouraged to corroborate the findings from this study, as well as to facilitate the triangulation of findings from varying methods and data sources to provide a comprehensive understanding of LTFU among TB patients. This may include analysis of quantitative data on determinants of LTFU among TB patients, as well as involving and incorporating experiences and perspectives from other parties (healthcare administrators or providers, caregivers, and family members of patients).

Additionally, we also propose that future TB control initiatives should look into exploring the implementation of innovative strategies in the monitoring and follow-up of TB treatment, including the use of digital health technologies. In 2015, WHO outlined a framework for digital health in the End TB strategy, with potential applications and innovative examples described in domains of patient care and electronic DOT (eDOT), surveillance monitoring, programmatic management and eLearning [44]. Further, recent studies have explored the utilisation of the Internet of Things (IoT) and artificial intelligence (AI) to augment digital health initiatives for TB control [45, 46]. In Malaysia, video-observed treatment (VOT) has been implemented in some healthcare facilities and is recommended as an alternative to DOT in selected patients where facilities are available [47]. Future research that explores and evaluates the feasibility, implementation and effectiveness of these initiatives should be encouraged.

## Conclusions

This qualitative study identified important contributory themes underlying the reasons for LTFU among TB patients in Malaysia. These included factors that occur at multiple levels, including patients’ personal level, healthcare system-related, structural factors as well as interpersonal level interaction. These factors, and the levels they occur at, represent issues and challenges faced by TB patients during their treatment duration and thus highlight areas where improvement and corrective actions can and should be targeted on. Integration of these qualitative findings into future policy considerations will help to inform the development and implementation of strategies that are responsive to TB patients’ needs and concerns, to effectively address LTFU and improve treatment completion rates among TB patients in Malaysia.

## Supporting information

S1 File Interview guide. (PDF)

S2 File Standards for Reporting Qualitative Research (SRQR) checklist. (PDF)

## References

[1] World Health Organization. Global Tuberculosis Report 2019. WHO/CDS/TB/2019.15. Geneva: World Health Organization; 2019.

[2] World Health Organization. The End TB Strategy. WHO/HTM/TB/2015.19. Geneva: World Health Organization; 2015.

[3] Disease Control Division (TB/Leprosy Sector) Ministry of Health Malaysia. National Strategic Plan for Tuberculosis Control (2016–2020). KKM/BI/5000/2018. Putrajaya: Ministry of Health Malaysia; 2016.

[4] Ministry of Health Malaysia. Clinical Practice Guidelines: Management of Tuberculosis (3rd edition). MOH/P/PAK/258.12(GU). Putrajaya: Ministry of Health Malaysia; 2012.

[5] Tok PSK, Liew SM, Wong LP, Razali A, Loganathan T, Chinna K, et al. Determinants of unsuccessful treatment outcomes and mortality among tuberculosis patients in Malaysia: A registry-based cohort study. PLoS One. 2020;15(4):e0231986. doi: 10.1371/journal.pone.0231986 32320443 PMC7176104

[6] World Health Organization. Definitions and reporting framework for tuberculosis (2013 revision, updated December 2014) WHO/HTM/TB/2013.2. Geneva: World Health Organization; 2013.

[7] World Health Organization. Adherence to long-term therapies: evidence for action. World Health Organization; 2003. https://apps.who.int/iris/handle/10665/42682

[8] da Silva Garrido M, Penna ML, Perez-Porcuna TM, de Souza AB, da Silva Marreiro L, Albuquerque BC, et al. Factors associated with tuberculosis treatment default in an endemic area of the Brazilian Amazon: a case control-study. PloS One. 2012;7(6):e39134. doi: 10.1371/journal.pone.0039134 22720052 PMC3373579

[9] Finlay A, Lancaster J, Holtz TH, Weyer K, Miranda A, van der Walt M. Patient-and provider-level risk factors associated with default from tuberculosis treatment, South Africa, 2002: a case-control study. BMC Public Health. 2012;12(1):56. doi: 10.1186/1471-2458-12-56 22264339 PMC3306745

[10] Gler M, Podewils L, Munez N, Galipot M, Quelapio M, Tupasi T. Impact of patient and program factors on default during treatment of multidrug-resistant tuberculosis. Int J Tuberc Lung Dis. 2012;16(7):955–60. doi: 10.5588/ijtld.11.0502 22584124 PMC4616015

[11] Kigozi G, Heunis C, Chikobvu P, Botha S, van Rensburg D. Factors influencing treatment default among tuberculosis patients in a high burden province of South Africa. Int J Infect Dis. 2017;54:95–102. doi: 10.1016/j.ijid.2016.11.407 27894985

[12] Lackey B, Seas C, Van der Stuyft P, Otero L. Patient characteristics associated with tuberculosis treatment default: A cohort study in a high-incidence area of Lima, Peru. PloS One. 2015;10(6):e0128541. doi: 10.1371/journal.pone.0128541 26046766 PMC4457855

[13] Lalor MK, Greig J, Allamuratova S, Althomsons S, Tigay Z, Khaemraev A, et al. Risk factors associated with default from multi-and extensively drug-resistant tuberculosis treatment, Uzbekistan: a retrospective cohort analysis. PloS One. 2013;8(11):e78364. doi: 10.1371/journal.pone.0078364 24223148 PMC3819387

[14] Park C-K, Shin H-J, Kim Y-I, Lim S-C, Yoon J-S, Kim Y-S, et al. Predictors of default from treatment for tuberculosis: a single center case–control study in Korea. J Korean Med Sci. 2016;31(2):254–60. doi: 10.3346/jkms.2016.31.2.254 26839480 PMC4729506

[15] Rutherford M, Hill P, Maharani W, Sampurno H, Ruslami R. Risk factors for treatment default among adult tuberculosis patients in Indonesia. Int J Tuberc Lung Dis. 2013;17(10):1304–9. doi: 10.5588/ijtld.13.0084 24025382

[16] Sitienei J, Kipruto H, Mansour O, Ndisha M, Hanson C, Wambu R, et al. Correlates of default from anti-tuberculosis treatment: a case study using Kenya’s electronic data system. Int J Tuberc Lung Dis. 2015;19(9):1051–6. doi: 10.5588/ijtld.14.0670 26260823

[17] Chida N, Ansari Z, Hussain H, Jaswal M, Symes S, Khan AJ, et al. Determinants of default from tuberculosis treatment among patients with drug-susceptible tuberculosis in Karachi, Pakistan: A mixed methods study. PLoS One. 2015;10(11):e0142384. doi: 10.1371/journal.pone.0142384 26562787 PMC4642974

[18] Dick J. The study of the determinants of non adherence to anti-tuberculosis treatment: are we using appropriate research methodology? Int J Tuberc Lung Dis. 1999;3(11):1049. 10587330

[19] Hasker E, Khodjikhanov M, Sayfiddinova S, Rasulova G, Yuldashova U, Uzakova G, et al. Why do tuberculosis patients default in Tashkent City, Uzbekistan? A qualitative study. Int J Tuberc Lung Dis. 2010;14(9):1132–9. 20819258

[20] Department of Statistics Malaysia. Population Distribution and Basic Demographic Characteristic Report 2010. Putrajaya: Department of Statistics Malaysia; 2011. Available from: https://tinyurl.com/dosm2010

[21] Gale NK, Heath G, Cameron E, Rashid S, Redwood S. Using the framework method for the analysis of qualitative data in multi-disciplinary health research. BMC Med Res Methodol. 2013;13(1):1–8. doi: 10.1186/1471-2288-13-117 24047204 PMC3848812

[22] Spencer L, Ritchie J. Qualitative data analysis for applied policy research. Analyzing qualitative data: Routledge; 2002. pp. 187–208.

[23] O’Brien BC, Harris IB, Beckman TJ, Reed DA, Cook DA. Standards for reporting qualitative research: a synthesis of recommendations. Acad Med. 2014;89(9):1245–1251. doi: 10.1097/ACM.0000000000000388 24979285

[24] Martins N, Grace J, Kelly PM. An ethnographic study of barriers to and enabling factors for tuberculosis treatment adherence in Timor Leste. Int J Tuberc Lung Dis. 2008;12(5):532–7. 18419889

[25] Munro SA, Lewin SA, Smith HJ, Engel ME, Fretheim A, Volmink J. Patient adherence to tuberculosis treatment: a systematic review of qualitative research. PLoS Med. 2007;4(7):e238. doi: 10.1371/journal.pmed.0040238 17676945 PMC1925126

[26] Muture BN, Keraka MN, Kimuu PK, Kabiru EW, Ombeka VO, Oguya F. Factors associated with default from treatment among tuberculosis patients in Nairobi province, Kenya: a case control study. BMC Public Health. 2011;11:696. doi: 10.1186/1471-2458-11-696 21906291 PMC3224095

[27] Schnaubelt E, Charles M, Richard M, Fitter D, Morose W, Cegielski J. Loss to follow-up among patients receiving anti-tuberculosis treatment, Haiti, 2011–2015. Public Health Action. 2018;8(4):154–61. doi: 10.5588/pha.18.0043 30775274 PMC6361484

[28] Chauke T, Netshikweta L, Netshandama V, Nyathi L, Tshitangano T, Olaniyi F. Proposed guidelines to minimise multi-drug resistant tuberculosis treatment default in a multi-drug resistant unit of Limpopo Province, South Africa. Afr J Infect Dis. 2018;12(2):55–65. doi: 10.21010/ajid.v12i2.9 30109287 PMC6085734

[29] Hane F, Thiam S, Fall AS, Vidal L, Diop AH, Ndir M, et al. Identifying barriers to effective tuberculosis control in Senegal: an anthropological approach. Int J Tuberc Lung Dis. 2007;11(5):539–43. 17439678

[30] Jaiswal A, Singh V, Ogden JA, Porter JD, Sharma PP, Sarin R, et al. Adherence to tuberculosis treatment: lessons from the urban setting of Delhi, India. Trop Med Int Health. 2003;8(7):625–33. doi: 10.1046/j.1365-3156.2003.01061.x 12828545

[31] Serapelwane MG, Davhana-Maselesele M, Masilo GM. Experiences of patients having tuberculosis (TB) regarding the use of Directly Observed Treatment Short-Course (DOTS) in the North West Province, South Africa. Curationis. 2016;39(1):e1–e9. doi: 10.4102/curationis.v39i1.1629 27796102 PMC6091630

[32] Reyes-Guillen I, Sanchez-Perez HJ, Cruz-Burguete J, Izaurieta-de Juan M. Anti-tuberculosis treatment defaulting: an analysis of perceptions and interactions in Chiapas, Mexico. Salud Publica Mex. 2008;50(3):251–7. doi: 10.1590/s0036-36342008000300009 18516373

[33] Sanchez-Padilla E, Marquer C, Kalon S, Qayyum S, Hayrapetyan A, Varaine F, et al. Reasons for defaulting from drug-resistant tuberculosis treatment in Armenia: a quantitative and qualitative study. Int J Tuberc Lung Dis. 2014;18(2):160–7. doi: 10.5588/ijtld.13.0369 24429307

[34] Cremers AL, Janssen S, Huson MA, Bikene G, Belard S, Gerrets RP, et al. Perceptions, health care seeking behaviour and implementation of a tuberculosis control programme in Lambarene, Gabon. Public Health Action. 2013;3(4):328–32. doi: 10.5588/pha.13.0038 26393056 PMC4463156

[35] Shiotani R, Hennink M. Socio-cultural influences on adherence to tuberculosis treatment in rural India. Glob Public Health. 2014;9(10):1239–51. doi: 10.1080/17441692.2014.953562 25223868

[36] Sripad A, Castedo J, Danford N, Zaha R, Freile C. Effects of Ecuador’s national monetary incentive program on adherence to treatment for drug-resistant tuberculosis. Int J Tuberc Lung Dis. 2014;18(1):44–8. doi: 10.5588/ijtld.13.0253 24365551

[37] Ukwaja K, Alobu I, Gidado M, Onazi O, Oshi D. Economic support intervention improves tuberculosis treatment outcomes in rural Nigeria. Int J Tuberc Lung Dis. 2017;21(5):564–70. doi: 10.5588/ijtld.16.0741 28399972

[38] Wei X, Zou G, Yin J, Walley J, Yang H, Kliner M, et al. Providing financial incentives to rural-to-urban tuberculosis migrants in Shanghai: an intervention study. Infect Dis Poverty. 2012;1(1):1–8. doi: 10.1186/2049-9957-1-9 23849348 PMC3710084

[39] Lutge EE, Wiysonge CS, Knight SE, Sinclair D, Volmink J. Incentives and enablers to improve adherence in tuberculosis. Cochrane Database Syst Rev. 2015(9):CD007952. doi: 10.1002/14651858.CD007952.pub3 26333525 PMC4563983

[40] Birch S, Govender V, Fried J, Eyles J, Daries V, Moshabela M, et al. Does treatment collection and observation each day keep the patient away? An analysis of the determinants of adherence among patients with Tuberculosis in South Africa. Health Policy Plan. 2016;31(4):454–61. doi: 10.1093/heapol/czv084 26384375

[41] Cherkaoui I, Sabouni R, Ghali I, Kizub D, Billioux AC, Bennani K, et al. Treatment default amongst patients with tuberculosis in urban Morocco: predicting and explaining default and post-default sputum smear and drug susceptibility results. PLoS One. 2014;9(4):e93574. doi: 10.1371/journal.pone.0093574 24699682 PMC3974736

[42] Malterud K. The art and science of clinical knowledge: evidence beyond measures and numbers. Lancet. 2001;358(9279):397–400. doi: 10.1016/S0140-6736(01)05548-9 11502338

[43] Kizub D, Ghali I, Sabouni R, Bourkadi JE, Bennani K, El Aouad R, et al. Qualitative study of perceived causes of tuberculosis treatment default among health care workers in Morocco. Int J Tuberc Lung Dis. 2012;16(9):1214–20. doi: 10.5588/ijtld.11.0626 22793783

[44] World Health Organization & European Respiratory Society. Digital health for the end TB strategy: an agenda for action. WHO/HTM/TB/2015.21. World Health Organization; 2015. https://apps.who.int/iris/handle/10665/205222

[45] Doshi R, Falzon D, Thomas BV, Temesgen Z, Sadasivan L, Migliori GB, et al. Tuberculosis control, and the where and why of artificial intelligence. ERJ Open Research. 2017;3(2):00056–2017. doi: 10.1183/23120541.00056-2017 28656130 PMC5478795

[46] Falzon D, Raviglione M. The Internet of Things to come: digital technologies and the End TB Strategy. BMJ Glob Health. 2016;1(2):e000038. doi: 10.1136/bmjgh-2016-000038 28588935 PMC5321338

[47] Ministry of Health Malaysia. Clinical Practice Guidelines: Management of Tuberculosis (Fourth Edition). MOH/P/PAK/469.21(GU)-e. Putrajaya: Ministry of Health Malaysia; 2021. Available from: https://www.moh.gov.my/moh/resources/Penerbitan/CPG/Respiratory/CPG-_Management_of_Tuberculosis_(4th_Edition).pdf.

