# Supplementary material for: A qualitative exploration of tuberculosis patients who were lost to follow-up in Malaysia
Source: PLoS One. 2023 Sep 7;18(9):e0289222. doi: 10.1371/journal.pone.0289222 (PMC10484432; doi:10.1371/journal.pone.0289222)
Supplement: S1 File — (PDF) [file pone.0289222.s001.pdf]

**Supplementary S1 File**

**INTERVIEW GUIDE**

Note: This serves as a guide for the interviews. The questions are in no particular order.

**Open-ended questions:**

1. What does tuberculosis or in short, TB, mean to you?

   *Prompt: Tell us what you know about TB/ about your understanding of TB.*

2. Could you describe your previous TB treatment experience?

3. Now we want you to think back to the time you stopped coming to healthcare facilities for TB treatment. Can you tell us what happened and/or what you did?

   *Prompt: Were there any difficulties or problems you faced during that time?*

   *Prompt: What contributed to you making the decision to not come anymore for the TB treatment?*

4. What do you think will happen to your health in the future?

5. We would like to hear more about your experience seeking treatment at healthcare facilities.

   *Prompt: Could you describe more on the:*

   - *Process of seeking treatment (time, logistics, organisation)*
   - *Healthcare workers that you meet (interaction, communication)*
   - *Did they affect you in any way?*

6. Has receiving TB treatment affected your life in any way? If yes, how?

   *Prompt: Relationships with family members, friends, at work or in school (if applicable)*

7. How is it like being in your community (neighbourhood) when you are taking TB treatment?

8. What do you think of the current TB treatment and follow-up for TB patients in Malaysia?

9. What improvements to the current TB treatment and follow-up in Malaysia do you suggest?

10. Is there anything else you would like to tell us about?

    *Prompt: Is there something we have missed that you think we should know?*
