# Supplementary material for: A qualitative exploration of tuberculosis patients who were lost to follow-up in Malaysia
Source: PLoS One. 2023 Sep 7;18(9):e0289222. doi: 10.1371/journal.pone.0289222 (PMC10484432; doi:10.1371/journal.pone.0289222)
Supplement: S2 File — (PDF) [file pone.0289222.s002.pdf]

Standards for Reporting Qualitative Research (SRQR)

O'Brien B.C., Harris, I.B., Beckman, T.J., Reed, D.A., & Cook, D.A. (2014). Standards for reporting qualitative research: a synthesis of recommendations. *Academic Medicine, 89(9)*, 1245-1251.

**No. Topic**	**Item**	**Description**
**Title and abstract**		
S1 Title	Concise description of the nature and topic of the study identifying the study as qualitative or indicating the approach (e.g., ethnography, grounded theory) or data collection methods (e.g., interview, focus group) is recommended	The title indicated 'qualitative exploration' of TB patients who were LTFU in Malaysia.
S2 Abstract	Summary of key elements of the study using the abstract format of the intended publication; typically includes objective, methods, results, and conclusions	Abstract provided, including the elements as outlined.
**Introduction**		
S3 Problem formulation	Description and significance of the problem/phenomenon studied; review of relevant theory and empirical work; problem statement	LTFU was reviewed, and the gap justifying this study was explained.
S4 Purpose or research question	Purpose of the study and specific objectives or questions	Objective was outlined at the end of the Introduction section.
**Methods**		
S5 Qualitative approach and research paradigm	Qualitative approach (e.g., ethnography, grounded theory, case study, phenomenology, narrative research) and guiding theory if appropriate; identifying the research paradigm (e.g., positivist, constructivist/interpretivist) is also recommended	The qualitative methods involved in this study were explained in the Materials and Methods section.

S6 Researcher characteristics and reflexivity	Researchers' characteristics that may influence the research, including personal attributes, qualifications/experience, relationship with participants, assumptions, or presuppositions; potential or actual interaction between researchers' characteristics and the research questions, approach, methods, results, or transferability	Materials and Methods (Reflexivity): Researcher background and reflexivity explained.
S7 Context	Setting/site and salient contextual factors; rationale[a]	Materials and Methods (Study population): The study setting/population was explained and justified.
S8 Sampling strategy	How and why research participants, documents, or events were selected; criteria for deciding when no further sampling was necessary (e.g., sampling saturation); rationale[a]	Materials and Methods (Sampling and recruitment): The sampling strategy was explained. It was also mentioned that participants' recruitment was until thematic saturation was reached.
S9 Ethical issues pertaining to human subjects	Documentation of approval by an appropriate ethics review board and participant consent, or explanation for lack thereof; other confidentiality and data security issues	Materials and Methods (Ethics consideration): Ethical considerations explained, the conduct of the study was approved by the Medical Research and Ethics Committee (MREC), Ministry of Health Malaysia.
S10 Data collection methods	Types of data collected; details of data collection procedures including (as appropriate) start and stop dates of data collection and analysis, iterative process, triangulation of sources/methods, and modification of procedures in response to evolving study findings; rationale[a]	Materials and Methods (Data collection): Data collection methods were described.
S11 Data collection instruments and technologies	Description of instruments (e.g., interview guides, questionnaires) and devices (e.g., audio recorders) used for data collection; if/how the instrument(s) changed over the course of the study	Semi-structured interview guides were used, this was explained under Materials and Methods (Data collection). S1 File – Interview guides.

S12 Units of study	Number and relevant characteristics of participants, documents, or events included in the study; level of participation (could be reported in results)	Study participants' characteristics were provided in Table 1.
S13 Data processing	Methods for processing data prior to and during analysis, including transcription, data entry, data management and security, verification of data integrity, data coding, and anonymization/deidentification of excerpts	Data processing procedures were explained in Materials and Methods (Data collection).
S14 Data analysis	Process by which inferences, themes, etc., were identified and developed, including researchers involved in data analysis; usually references a specific paradigm or approach; rationale[a]	Materials and Methods (Data analysis and reporting): Framework method was used for data analysis; explained.
S15 Techniques to enhance trustworthiness	Techniques to enhance trustworthiness and credibility of data analysis (e.g., member checking, audit trail, triangulation); rationale[a]	Materials and Methods (Reflexivity): It was mentioned and explained how other investigators were involved.
**Results/Findings**		
S16 Synthesis and interpretation	Main findings (e.g., interpretations, inferences, and themes); might include development of a theory or model, or integration with prior research or theory	Results section: Table 2 illustrates the main findings (themes) of the study.
S17 Links to empirical data	Evidence (e.g., quotes, field notes, text excerpts, photographs) to substantiate analytic findings	Results section: In each of the themes/factors analysed, text excerpts were provided.
**Discussion**		
S18 Integration with prior work, implications, transferability, and contribution(s) to the field	A short summary of main findings; explanation of how findings and conclusions connect to, support, elaborate on, or challenge conclusions of earlier scholarship; discussion of scope of application/generalizability; identification of unique contribution(s) to scholarship in a discipline or field	Findings were summarized, and discussed.

S19 Limitations	Trustworthiness and limitations of findings	Limitations of findings were mentioned and discussed.
**Other**		
S20 Conflicts of interest	Potential sources of influence or perceived influence on study conduct and conclusions; how these were managed	“The authors declare no conflict(s) of interest”.
S21 Funding	Sources of funding and other support; role of funders in data collection, interpretation, and reporting	“No funding received for this study.”

[a]The rationale should briefly discuss the justification for choosing that theory, approach, method, or technique rather than other options available, the assumptions and limitations implicit in those choices, and how those choices influence study conclusions and transferability. As appropriate, the rationale for several items might be discussed together.
